# Does the Internet Expand the Educational Gap Among Different Social Classes? The Protective Role of Future Orientation

**DOI:** 10.3389/fpsyg.2021.647351

**Published:** 2021-05-04

**Authors:** Jing-Jing Chen, Ming Fei Liu

**Affiliations:** ^1^Department of Sociology, Faculty of Humanities and Social Sciences, Nanjing Forestry University, Nanjing, China; ^2^Nanjing Zhonghua High School, Nanjing, China

**Keywords:** family socioeconomic status, future orientation, internet usage preference for entertainment, academic achievement, moderated mediation model

## Abstract

Amid the social background of China where the Internet has penetrated into every corner of an adolescent’s life, we were concerned of the role of Internet usage in influencing the educational gap among social classes. We investigated the mediating role of Internet usage preference for entertainment in the relationship between the family socioeconomic status (SES) and the adolescent’s academic achievement and explored the moderating role of future orientation in the relationship. A total of 614 junior high school students were recruited to complete a questionnaire survey, including questionnaires for family SES, Internet usage preference, and adolescent future orientation. The results showed that (1) the relationship between family SES and academic achievement was mediated by Internet usage preference for entertainment; (2) the indirect effect was moderated by future orientation, such that the negative association between family SES and Internet usage preference for entertainment was only indicated in adolescents with low future orientation; and (3) the direct association between family SES and Internet usage preference for entertainment was moderated by future orientation.

## Introduction

Socioeconomic status (SES) is used to describe and rank valuable resources (such as education, wealth, and social status, etc.) owned by families (Matthews and Gallo, 2011). Previous studies have shown that children from families with higher SES are more likely to achieve academic success, while children from families of lower SES have more problems with academic performance and achievement (Barr, 2015; Li et al., 2020). The relationship between family background and educational achievement is one of the important indicators to measure educational equity (Li and Qiu, 2018). In this context, it is deemed important to explore the influence mechanism of family SES on adolescents’ academic achievement and discuss the corresponding intervention strategies.

### The Mediating Role of Internet Usage Preference for Entertainment

In recent years, with the rapid development of information technology and the rapid popularization of the Internet, the use of information technology to achieve a higher level of education equity has become an established national policy in China. “The Outline of National Medium and Long-Term Educational Reform and Development (2010–2020)” clearly points out to “vigorously promote education equity. build an effective mechanism to expand the coverage of high-quality education resources by means of information technology and gradually narrow the gap among different regions, urban-rural and inter-schools.” Given the vigorous implementation of relevant policies, the Internet coverage in China has expanded. However, the gap in education among different regions and social classes has not been narrowed. More and more scholars have shifted the focus of the problem from the “physical gap” between social classes, that is, the penetration rate of the Internet, to the “usage gap” of the Internet (Wang and Sui, 2014).

Previous studies have found that individuals with high SES use more “serious applications” such as education or information acquisition to maximize the advantage effect of the Internet on capital and resources related to work, study, and social participation, while individuals with low SES mainly use “entertainment applications” such as chat and online games and lack the willingness and motivation to find information with educational value (Van Dijk, 2012; Chen, 2013). If adolescents of different SES have equal access to the Internet, but those with low SES only use the Internet to make friends, play games, and watch videos, and because of the lack of meaningful learning activities such as scientific information exploration and problem solving, it can be expected that the Internet will only expand the educational gap among social classes. At present, there is no empirical study to explore that families with low SES will negatively affect adolescents’ academic performance by promoting adolescents to form entertainment-oriented Internet use. This hypothesis is supported by the cultural reproduction theory, according to which individuals of different social classes inherit different cultural capital from their families, such as ways of thinking and living, beliefs, hobbies, and interests, which in turn affect the academic performance of adolescents from different social classes (Lamont and Lareau, 1988). Families with low SES usually lack a cultural atmosphere of learning, exploration, and reading, and adolescents themselves are limited in their literacy interests (Van Vechten, 2013). Meanwhile, adolescents of low SES usually have to spend much more time at home, lack resources for meaningful structured after-school activities, and use digital devices as cheap alternatives for filling free time (Faltýnková et al., 2020). Against this background, adolescents from low SES are more likely to form entertainment network preferences. Thus, the Internet has not become an opportunity for adolescents from low SES to learn new knowledge and acquire new skills. On the contrary, it may take up more learning time and lead to a decline in their academic performance (Zhang and Huang, 2018).

### The Moderating Role of Future Orientation

Although the predictive effect of family SES on adolescents’ academic achievement has been verified by many previous studies, there is still a phenomenon whereby “poor families produce noble children” in real life. In such cases, the theory of resilience provides an analytical perspective. The resilience theory is used to analyze the phenomenon whereby disadvantaged individuals still achieve good adaptation (Masten, 2014). This theory posits that the psychological condition of disadvantaged individuals is the result of the combined effect of many risk factors and protective factors in their environment. If children have protective factors to deal with the adverse situation, they may still maintain normal development, and even their development level will exceed the development levels of their peers (Luthar et al., 2000). Based on this theoretical perspective, it is worthwhile to identify the protective factors for children from low family SES to obtain academic success. Previous studies have verified that certain individual characteristic variables, such as individual learning motivation, can reduce the influence of family SES on adolescents’ academic achievement (Chen et al., 2018). Future orientation is used to examine individuals’ thinking and planning toward the future (Nurmi, 1991; Liu et al., 2010). A few studies pointed out that an individual’s belief in goal realization and an optimistic attitude toward the future also act as protective factors that help disadvantaged individuals overcome difficulties and obtain adaptive results (Yu and Han, 2018). Accordingly, this study explores the protective effect of future orientation on academic success of adolescents with low family SES, and it speculates that future orientation can regulate the relationship between family SES and academic achievement.

This study also speculates that future orientation can alleviate or block the promotion of low family SES on Internet usage preferences for entertainment and indirectly have a protective effect on adolescents’ academic achievement. Previous research results show that individuals with high-level future orientation, which means one can look forward to the future with a long-term perspective, set clear goals, and have a more optimistic attitude for the future, can take more goal-oriented behaviors and improve their academic performance by delaying gratification (Bembenutty and Karabenick, 2004; Du and Lv, 2017). At the same time, individuals who can see the long-term results of current behaviors are more likely to avoid socially non-adaptive behaviors such as drug abuse (Breier-Williford and Bramlett, 1995; Zhang et al., 2018). These previous results suggest that a high-level future orientation will help individuals resist the temptation of Internet entertainment information and reduce non-adaptive online behaviors.

### The Present Study

**FIGURE 1:**
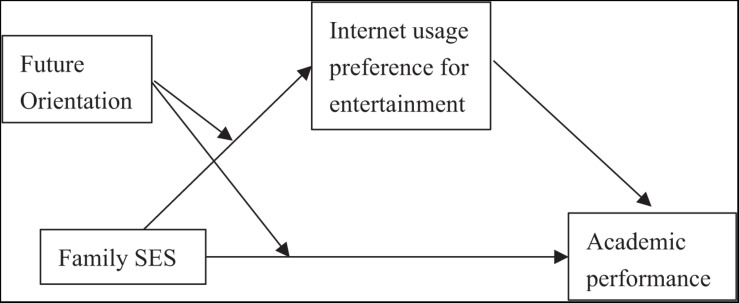
A hypothesized moderated mediation model.

To sum up, this study takes junior high school students as the research subjects and builds a moderated mediation model based on the perspective of cultural reproduction theory and resilience theory (Figure 1). The aim is to explore the mediating (Internet entertainment usage preference) and moderator (future orientation) mechanisms of family SES in predicting academic achievement, so as to clarify the internal influence mechanism of family SES on adolescents’ academic achievement, while providing theoretical and practical support for the realization of educational equity among different social classes.

## Materials and Methods

### Participants

The convenience sampling method was used to select research schools. Data were collected from a public junior high school in Nanjing, Jiangsu Province. Considering that junior high school students facing entrance examinations are banned or have less time to use the entertainment functions of the Internet, a questionnaire survey was conducted only between the first and second grades using the cluster sampling method based on class as the unit. A total of 650 questionnaires were distributed, and large areas of blank questionnaires and questionnaires that were obviously filled in randomly were eliminated. Finally, 614 valid questionnaires were obtained, with an effective rate of 94.46%. In the valid questionnaires, there were 314 students (51.14%) in the first grade and 300 students in the second grade (48.86%); in addition, there were 274 boys (44.79%) and 340 girls (55.37%). The average age was 13.6 years, and the standard deviation was 0.71. For a small number of questionnaires with missing values, the average value of the variable was used to replace the missing value.

### Procedure

The questionnaires are presented in the order of Internet usage preference scale, adolescent future orientation scale, questionnaire for family SES, and examination scores and some other demographic variable information. The questionnaire survey was conducted using part of class time. Permission from school leaders, students, and their parents was obtained prior to administering the questionnaire. Before filling in the questionnaire, all participants were informed of the anonymity of the questionnaire and matters unrelated to school academic performance, and students are required to complete the questionnaire independently. All the questionnaires were collected on the spot.

### Variables Measured

#### Family SES

Referring to the relevant literature (Bradley and Corwy, 2002; Ren, 2010), family SES was measured by three indicators: family monthly income, father and mother’s education level, and father and mother’s occupation. Eleven options from “less than 2,000 yuan” to “more than 20,000 yuan” were set for family monthly income, with 1–11 points from low to high. Six options from “primary school or below” to “postgraduate (master’s or doctor’s degree)” were set for parents’ education, with the same value of one point to six points from low to high. For the division of parents’ occupations, reference was made to relevant studies such as Xia et al. (2017), who proposed 10 options from “urban and rural unemployment” to “state and social managers” and in turn assigned one point to 10 points. The higher the score, the higher the professional social status. Referring to previous studies (Xia et al., 2017), the three indicators of parents’ occupation level, parents’ education level, and family monthly income were converted into standard scores, and principal component analysis was conducted. The results showed that a principal factor with a characteristic root greater than 1 explained 59.73% of the variance. Accordingly, we only need to present the coefficient of main factor 1 to get the calculation formula of family comprehensive SES index, that is, family SES = (0.793 ^∗^ *Z* parents’ occupation + 0.779 ^∗^ *Z* family monthly income + 0.746 ^∗^ *Z* parents’ education level)/1.792. Among them, 0.793, 0.779, and 0.746 are the factor loading of the three indicators, respectively, and 1.792 is the characteristic root of the first factor. The range of family SES is between −3.60 and 2.14. The higher the value, the higher the individual’s family SES.

#### Academic Achievement

Academic achievement was measured by students’ scores in the three subjects of Chinese, mathematics, and English in the most recent grade unified examination. The average scores of the three test subjects were calculated and then converted respectively into standard scores within the grade. Higher values indicated a higher level of the individual’s academic achievement.

#### Internet Usage Preference for Entertainment

The Internet usage preference inventory for adolescents was invented by Chen et al. (2010). In this study, we only use the subinventory of entertainment preference. The subinventory contains six items, an example of which is “Watch Internet TV and movies, etc.” Participants were asked to rate each item from 1 (rarely use) to 5 (always use). The higher the value, the more often the individual uses entertainment functions and information on the Internet. Cronbach’s alpha was 0.76 in the present study.

#### Future Orientation

Future orientation was measured by a Chinese version of the Future Orientation Scale invented by Liu et al. (2011). This scale contains 31 items, and one of the examples is “I often think about things to do in the future.” Participants rated each item on a five-point scale ranging from “1 = completely inconsistent” to “completely consistent.” The average of 31 items was calculated, with a higher score indicating greater capacity to perceive future time as expansive and optimistic. Cronbach’s alpha was 0.90 in the present study.

### Statistical Analyses

Data analyses were performed using SPSS 19.0 and the PROCESS macro for SPSS (Hayes, 2013). First, we conduct analyses of descriptive statistics and Pearson correlation analysis to have a preliminary overview of study variables. Second, we used PROCESS 3.3 to run the mediation and moderated mediation analyses using Model 8. Direct and indirect effects were estimated using Preacher and Hayes’ bias-corrected non-parametric bootstrapping techniques with 5,000 bootstrap samples (Preacher and Hayes, 2004). As suggested by prior research (Settanni et al., 2018), the existence of mediation and moderated mediation effects was further evaluated using 95% bias-corrected CIs. If the confidence intervals did not contain zero, these effects were considered statistically significant.

## Results

### Assessing of Common Method Bias

In this study, procedural control and statistical testing were used to reduce and verify the common method bias problem. In terms of procedural control, by informing the subjects of the anonymity and confidentiality of the questionnaire, the items of the same dimension were arranged in disorder and the reverse items were set in the questionnaire to reduce the common method bias. In addition, Harman’s single-factor test was used for the collected data to assess the common method bias, that is, an unrotated exploratory factor analysis is performed on all measurement items. The results showed that there are 10 factors with eigenvalues greater than 1, and the variance explanation rate of the first factor is 23.18%, which is less than the critical standard of 40%. It can be judged that there is no serious common method bias problem in the data of this study.

### Preliminary Analyses

Means and standard deviations for study variables and bivariate correlation are reported in Table 1. As the table shows, family SES was positively correlated with academic achievement but not with future orientation; future orientation is significantly positively correlated with academic achievement but negatively correlated with Internet usage preference for entertainment; academic achievement is significantly negatively correlated with Internet usage preference for entertainment.

**TABLE 1:** Descriptive statistics and intercorrelations between study variables (*N* = 614).

	*M*	SD	1	2	3	4	5
1. Family SES	0.00	1.00	1	0.035	0.132**	–0.042	0.010
2. Future orientation	3.60	0.52		1	0.160**	−0.112**	0.053
3. Academic achievement	0.00	0.89			1	−0.097*	0.029
4. Internet usage preference for entertainment	2.94	0.82				1	–0.009
5. Gender							1

*p < 0.05; **p < 0.01; and ***p < 0.001.

### Testing for a Moderated Mediation Model

According to the test method of the moderated mediation model suggested by previous research (Baron and Kenny, 1986; Wen and Ye, 2014), the following three regression equations were constructed, in which *X*, *Y*, *M*_*o*_, and *M*_*e*_ represent family SES, academic achievement, future orientation, and Internet usage preferences for entertainment, respectively. All variable values are converted into standard scores. The parameters of the three regression equations were tested. If *a*_1_ or *a*_3_ and *b*_1_ are significant, the moderated mediating model is satisfied.

(1) Y=c+0cX1+cM2+ocXM3+oe1

(2) M=ea+0aX1+aM2+oaXM3+oe2

(3) Y=c+0′cX1′+cM2′+ocXM3′+obM1+ee3

Path coefficients of the moderated mediation model are reported in Table 2. In Equation 1, family SES was significantly related to academic achievement (β = 0.127, *p* < 0.001); the interaction between family SES and future orientation was significantly linked to academic achievement (β = 0.083, *p* < 0.05). In Equation 2, the path coefficient of family SES on Internet usage preference for entertainment is not significant; the interaction between family SES and future orientation was significantly linked to Internet usage preference for entertainment (β = 0.090, *p* < 0.05). In Equation 3, both family SES (β = 0.125, *p* < 0.01) and Internet usage preference for entertainment (β = *−*0.085, *p* < 0.05) were significantly linked to academic achievement; the interaction between family SES and future orientation was significantly linked to academic achievement (β = 0.090, *p* < 0.01). Based on the above results, the moderated mediation model proposed in this study was supported.

**TABLE 2:** Parameter estimates of research models (*N* = 596).

	Equation 1		Equation 2		Equation 3	
			
	(DV: academic achievement)		(DV: entertainment)		(DV: academic achievement)	
			
	β	*t*	β	*t*	β	*t*
Family SES	0.127***	3.192	–0.039	–0.835	0.125**	3.128
Future orientation	0.188***	4.618	−0.095*	–2.287	0.180***	4.414
Entertainment					−0.085*	–2.099
Family SES * future orientation	0.083*	2.218	0.088*	2.307	0.090**	2.413
*R*^2^	0.057		0.021		0.064	
*F*	11.960***		4.182**		10.123***	

*p < 0.05; **p < 0.01; and ***p < 0.001; entertainment is short for internet usage preference for entertainment.

**FIGURE 2:**
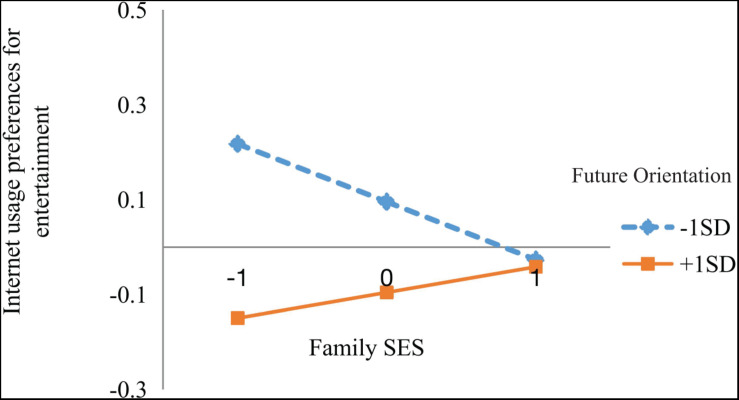
Future orientation as a moderator of the direct relationship between SES and entertainment usage.

**FIGURE 3:**
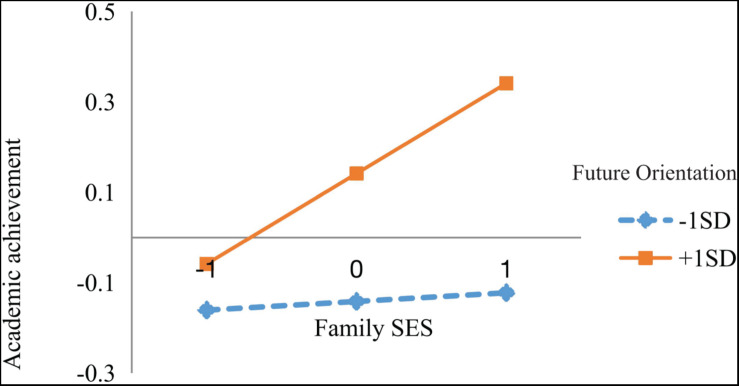
Future orientation as a moderator of the direct relationship between SES and academic achievement.

The nature of the significant interaction effect was examined by a simple slope analysis. As shown in Figures 2, 3, future orientation was divided into two levels based on mean (low = *M* − 1 SD, high = *M* + 1 SD). The association between family SES and Internet entertainment usage preference was significant only for individuals with a low level of future orientation (β = −0.120, *p* < 0.05), but not for those with a high level of future orientation (β = 0.054, *p* = 0.330) (Figure 2). The bias-corrected centile bootstrap method was used for the mediation test. The mediating effect at a high level of future orientation was −0.005 [95% CI (−0.019, 0.007)], and at a low level of future orientation, it was 0.010 [95% CI (0.002, 0.025)]. Only the mediating effect at a high level of future orientation did not include zero in terms of 95% confidence intervals. In other words, the mediating effect of family SES on academic achievement through Internet entertainment preference is moderated by future orientation.

Additionally, the direct effects of family SES on academic performance were moderated by future orientation. The results of the simple slope test showed that the association between family SES and academic achievement was significant only for individuals with higher levels of future orientation (*M* + 1SD) (β = 0.215, *p* < 0.001), but not for those with lower levels of future orientation (*M* − 1SD) (β = 0.036, *p* = 0.664) (Figure 3).

## Discussion

Exploring the association between family SES and academic performance has always been an important subject of educational equality. In the current Chinese society where the Internet has gradually penetrated into the daily lives of adolescents and has an important impact on their psychological development, the present study took junior high school students as subjects, used Internet usage preference for entertainment as a mediator variable, explored the influential mechanism of family SES on adolescents’ academic achievement, and examined the moderating effect of future orientation as a protective factor. The results showed that the effect of family SES on academic achievement is mediated by Internet usage preference for entertainment, and this effect is moderated by adolescent’s future orientation.

### The Relationship Between Family SES, Internet Usage Preference for Entertainment, and Academic Achievement

This study showed that family SES positively predicts academic achievement, which is consistent with previous conclusions (Barr, 2015; Li et al., 2020). Existing theories including the family investment theory (Haveman and Wolfe, 1994), social cognitive theory (Kraus et al., 2012), and cultural psychology theory (Grossmann and Varnum, 2011) explain the relationship between family SES and individual academic achievement from different perspectives. Guo et al. (2017) summarized that the differences in the possession of resources in families of different social classes, the differences in the abilities and personality traits (such as self-efficacy, etc.) of individuals in different social classes, and the degree of overlap between school culture and culture of different classes all effectively explained that people with low socioeconomic status are more difficult to achieve academic success.

This study also found that Internet entertainment usage preference negatively predicts academic achievement, which was also consistent with previous researches (Kirschner and Karpinski, 2010; Junco, 2011). Compared with academic activities that require abstract thinking skills, the pictures, animations, film images in online media, and virtual images in games are more attractive to adolescents. However, long-term games and animation stimulation will not only take up the time required for intellectual development and learning of adolescents but also affect the cognitive development of adolescents and reduce students’ ability to read and understand text and their interest in learning (Mössle et al., 2010). Additionally, some scholars pointed out that entertainment information such as games and animation has the characteristics of high arousal and rapid presentation (Schmidt and Anderson, 2007). Long-term immersion in this kind of stimulus will reduce the attention span and duration of adolescents (Ophir et al., 2009). It is difficult to maintain continuous attention in a low-arousal environment like the classroom, which has a negative impact on their academic performance (Wu, 2016).

There is no significant correlation between family SES and Internet entertainment usage preference, but this finding is not consistent with previous research conclusions (Van Dijk, 2012; Chen and Gu, 2017). Previous studies based on the theory of cultural reproduction have asserted that children from low SES families are more likely to form entertainment preferences (Zhang and Huang, 2018). However, many studies have pointed out that although cultural reproduction theory has a certain explanatory power, it overemphasizes the decisive role of external social structure on family reproduction, ignoring the performance of individual initiative (Yu and Han, 2018) or internal drive (Zhu, 2018). In other words, there may be many protective factors of family characteristics or individual personality traits which moderate the association between family SES and academic-related non-adaptive behavior (Li, 2009). These protective factors, such as future orientation discussed in this study, can hinder adolescents from low SES families from forming Internet entertainment usage preferences, leaving no clear relationship between the two.

### The Influence Mechanism of Family SES on Academic Achievement: Moderated Mediating Effect

The results of this study indicate that Internet entertainment usage preference has as a mediator role in the relationship between family SES and academic achievement, but this mediating effect is only reflected in the group with a low level of future orientation. In other words, in the group with a low level of future orientation, the lower the family’s SES, the more personal entertainment-oriented Internet usage behaviors will be, which will negatively affect their academic achievements. Meanwhile, Internet entertainment usage preference has no significant mediating effect on the relationship between family SES and academic achievement in the group with a high level of future orientation.

The need satisfaction theory points out the reason why individuals use certain network functions and form specific network behavior preferences is that some potential needs are ignored in real life, but can be awakened and satisfied in the Internet world (John, 1999). Parents of low SES families tend to spend less time with their children and are more likely to adopt negative parenting styles such as apathy and punishment (Zhang et al., 2017). In such a family environment, adolescents’ needs such as intimacy, belonging, and achievement cannot be met, so they are more likely to seek satisfaction from the Internet through making friends on the Internet. Especially in the case of low level of future orientation, the lower the SES of individuals, the more likely they are to form Internet usage preference for entertainment. However, based on the results of this study, it can be seen that as the individual’s development level of future orientation increases, such influence relationships will change. In the group with a high level of future orientation, the influence of family SES on Internet entertainment usage preference is not significant, which indicates that future orientation is a protective factor for adolescents from low SES families to form Internet entertainment usage preference. Previous studies have pointed out that a high level of future orientation means that individuals are more inclined to see the long-term consequences of current behaviors, which can reduce the use of drugs, academic procrastination, and other social non-adaptive behaviors (Zimbardo and Boyd, 1999; Yang et al., 2020). Individuals with a high level of future orientation can see the long-term harm of indulging in Internet entertainment information, so they can use the Internet more sparingly and avoid the decline of academic performance.

The results also show that future orientation moderates the direct impact of family SES on academic achievement. In the group with a high level of future orientation, family SES has a positive predictive effect on academic achievement, while in the group with a low level of future orientation, the influence of family SES on academic achievement is not significant. In other words, the high level of future orientation strengthens the positive predictive effect of family SES on academic achievement. Family SES mainly reflects the actual possession of cultural and economic resources. Some scholars point out that when considering the influence of the family’s economic and cultural environment on the growth of adolescents, it is necessary to distinguish between owning capital and activating capital (Wang and Ma, 2019). Previous studies have pointed out that the objective economic and cultural resources of the family cannot directly predict the academic achievements of their children. Only when the high SES of the family, parents’ participation, and so on become a promoting factor for the formation of adolescents’ educational values can these have a positive predictive effect on the adolescents’ academic achievements (Yang and Wan, 2015; Chen and Xu, 2020). Individuals with a high level of future orientation can see the value of education in a long time span, so they may be more active in transforming the family’s economic and cultural resources into resource advantages for academic success.

### Practical Implications and Limitations

The findings of this study provide practical insights to improve the academic performance of adolescents from low SES. Schools need to exercise supervision and management in order to encourage the socially disadvantaged adolescents to realize the value of Internet technology for their own learning and development while providing compensatory guidance on Internet use. Meanwhile, parents should manage and guide their children’s Internet usage behavior to prevent them from being overly addicted to online entertainment information.

Additionally, improving the development level of adolescent’s future orientation can not only prevent adolescents of low SES from forming Internet entertainment usage preference, but also enhance the positive predictive effect of the family SES on the academic achievements of adolescents. Parents should encourage and support their children to explore their future, guide them to construct future goals and plans, and encourage them to implement plans. On the other hand, previous studies have pointed out that teachers can help socially disadvantaged adolescents to recognize the long-term value of school education, and help raise their educational expectations to improve their academic motivation (Wang and Ma, 2019). Therefore, teachers’ attention and encouragement will promote the development of the future orientation of socially disadvantaged adolescents.

Finally, some limitations of this study should be acknowledged. First, the present study used a cross-sectional design, which is not conducive to clarifying the causal relationship between variables. Longitudinal tracking should be further used to examine the dynamic relationship between family SES, Internet usage preference, and academic performance. Second, this study used observed variables in statistical analysis. The results may be more accurate if latent variables are used.

## Conclusion

This study explored the internal influence mechanism of family SES on the educational achievements of adolescents from the perspective of Internet usage preference and verified the protective effect of future orientation. The results of the present study expanded upon the existing results about the relationship between social class and educational gaps and provided intervention insight for interfering with the low educational achievements of adolescents from lower SES. A high-level future orientation can not only reduce the use of the entertainment function of the Internet and thus avoid the decline in academic performance, but also enable adolescents to actively transform the family’s economic and cultural resources into their own resource advantages for academic success. Therefore, in future researches, we can try to improve the future orientation of adolescents from low SES, to help them improve their academic performance, so as to achieve upward movement of social class.

## Data Availability Statement

The raw data supporting the conclusions of this article will be made available by the authors, without undue reservation.

## Ethics Statement

Ethical review and approval was not required for the study on human participants in accordance with the Local Legislation and Institutional requirements. Written informed consent to participate in this study was provided by the participants’ legal guardian/next of kin.

## Author Contributions

J-JC designed the framework of this research, analyzed the data, and wrote the manuscript. ML assisted in the investigation. Both authors contributed to the article and approved the submitted version.

## Conflict of Interest

The authors declare that the research was conducted in the absence of any commercial or financial relationships that could be construed as a potential conflict of interest.
